# De Novo Lupus Anticoagulant-Positive Catastrophic Antiphospholipid Syndrome With Multiorgan Microvascular Failure: A Thrombotic Cascade Beyond Control

**DOI:** 10.7759/cureus.113799

**Published:** 2026-08-02

**Authors:** Mohd Laraib, Anoop Tiwari, Kamal Saini, Preeti Solanki, Satish Kumar, Payal Jain

**Affiliations:** 1 Department of General Medicine, Government Institute of Medical Sciences, Greater Noida, IND

**Keywords:** catastrophic antiphospholipid syndrome, lupus anticoagulant, microvascular thrombosis, multiorgan failure, thrombotic microangiopathy

## Abstract

Catastrophic antiphospholipid syndrome (CAPS) is an uncommon but life-threatening manifestation of antiphospholipid syndrome characterized by rapidly progressive multiorgan failure due to diffuse small-vessel thrombosis. Early recognition is challenging, particularly in patients without prior obstetric morbidity or established autoimmune disease. We report a 38-year-old woman who presented with acute limb ischemia, respiratory distress, thrombocytopenia, and acute cerebral infarcts. Laboratory evaluation revealed lupus anticoagulant positivity, reduced protein C, protein S, and antithrombin III levels, and elevated D-dimer. Imaging demonstrated diffuse distal arterial narrowing without large-vessel occlusion, bilateral pulmonary ground-glass opacities, and multiple lacunar infarcts. Skin biopsy showed occlusive arteriolopathy without evidence of vasculitis, and direct immunofluorescence was negative. A heterozygous factor V Leiden mutation was detected. A diagnosis of microvascular thrombotic disease consistent with CAPS was made after the exclusion of vasculitis and embolic causes. Despite prompt anticoagulation and corticosteroid therapy, she developed progressive multiorgan dysfunction and died. This case highlights the importance of recognizing CAPS as a predominantly microvascular thrombotic disorder that may present without prior features of antiphospholipid syndrome. The coexistence of inherited thrombophilia may act as a prothrombotic amplifier and contribute to a fulminant clinical course.

## Introduction

Antiphospholipid syndrome (APS) is an autoimmune thrombotic disorder that may occur as a primary condition or secondary to other autoimmune diseases, most commonly systemic lupus erythematosus (SLE). APS is defined by arterial or venous thrombosis and/or pregnancy morbidity in the presence of antiphospholipid antibodies [1]. Catastrophic antiphospholipid syndrome (CAPS) represents the most severe end of this spectrum and is characterized by rapidly progressive small-vessel thrombosis leading to multiorgan failure over days [2]. Although it occurs in fewer than 1% of patients with APS, mortality remains high despite treatment [2,3]. CAPS is frequently triggered by infection, surgery, or inflammatory stress and is thought to result from an exaggerated thromboinflammatory response in predisposed individuals [2].

Recognition may be difficult in patients without previous obstetric morbidity or documented APS. We describe a patient with acute multiorgan microvascular thrombosis and lupus anticoagulant positivity in whom CAPS was diagnosed after the exclusion of alternative causes.

## Case presentation

**Figure 1:**
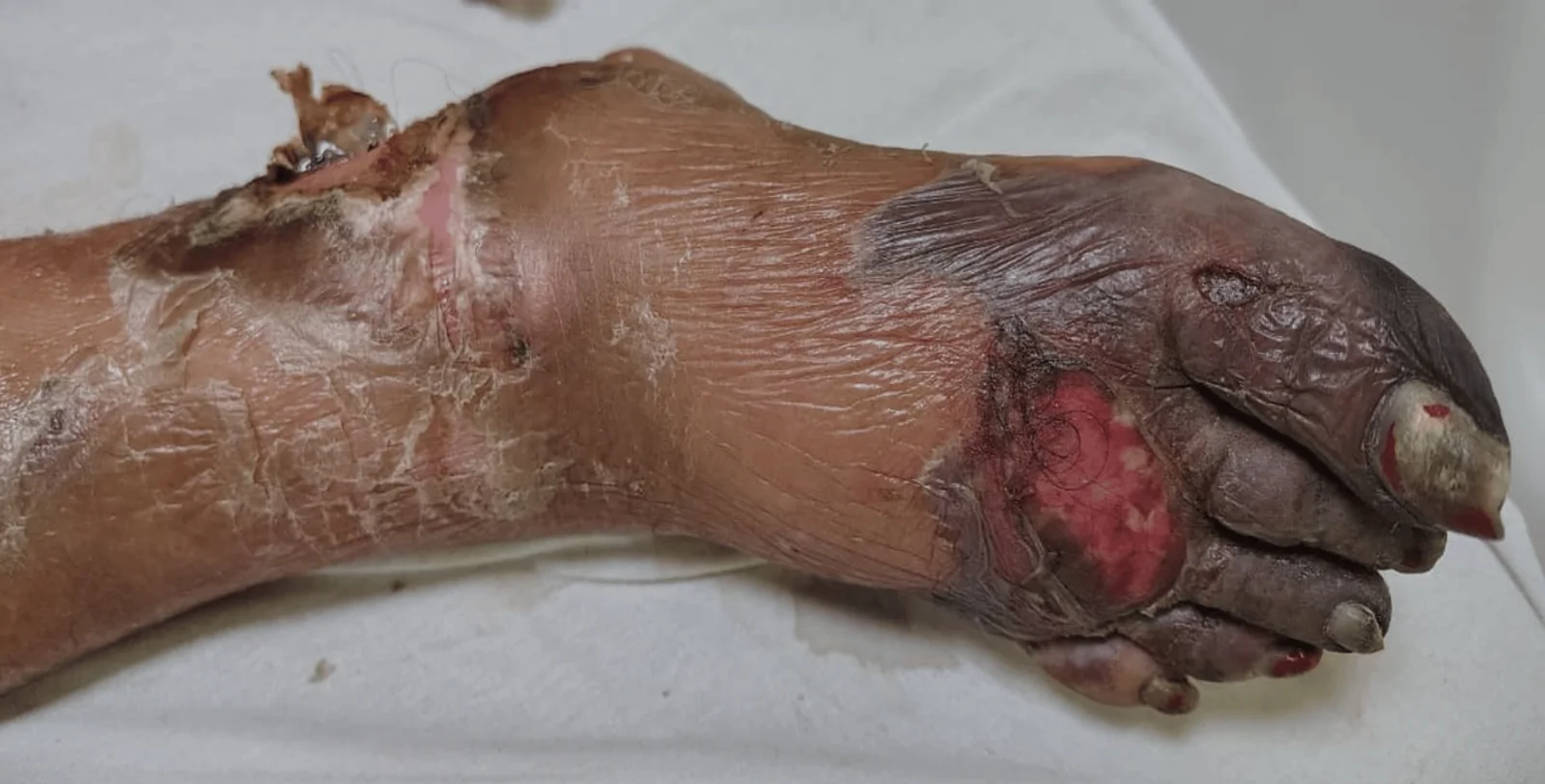
Clinical photograph showing acute ischemic gangrene of the patient's right foot

A 38-year-old woman with no known comorbidities presented with an acute onset of severe pain in both lower limbs with bluish discoloration of the fingers of the right foot for three days (Figure 1), accompanied by rapidly progressive dyspnea and tachypnea, reduced urine output, and altered sensorium. There is no history of chest pain, palpitations, or fever at symptom onset, trauma, or recent immobilization. She also had a painful ulcer over the medial malleolus of 7-10 days' duration. She had previous uncomplicated term pregnancies without recurrent pregnancy loss, intrauterine fetal demise, preeclampsia, or other obstetric manifestations of APS and no prior history of digital gangrene, chronic non-healing ulcers, or limb ischemia. She had no history of oral contraceptive pill use, hormonal therapy, recent pregnancy or postpartum state, nonsteroidal anti-inflammatory drug use, corticosteroid use, herbal or alternative medication intake, or prior exposure to anticoagulants.

A detailed systemic review was negative for features suggestive of connective tissue disease or systemic vasculitis, including photosensitivity, oral or nasal ulcers, alopecia, inflammatory arthritis, serositis (pleuritic chest pain or pericardial pain), or Raynaud phenomenon. There were no constitutional symptoms such as prolonged fever, weight loss, anorexia, or night sweats to suggest underlying malignancy or chronic infection. There was no history of diarrhea, vomiting, respiratory tract infection, urinary tract infection, recent vaccination, or other symptoms suggestive of an acute infectious illness.

Laboratory evaluation demonstrated thrombocytopenia with a declining trend, normocytic anemia, a normal activated partial thromboplastin time, elevated D-dimer levels, reduced fibrinogen, elevated inflammatory markers, elevated troponin I, and acute kidney injury. Lupus anticoagulant was positive by dilute Russell viper venom testing. Protein C, protein S, and antithrombin III levels were reduced. Serum complement levels revealed reduced C3 and C4. Antinuclear antibody and antineutrophil cytoplasmic antibodies were negative. Anti-β2 glycoprotein I IgA was elevated. Genetic testing revealed a heterozygous factor V Leiden mutation, suggesting a prothrombotic second-hit mechanism. The patient's laboratory investigations are summarized in Table 1.

**Table 1:** Laboratory investigations at presentation ALT: alanine aminotransferase; AST: aspartate aminotransferase; PT: prothrombin time; INR: international normalized ratio; APTT: activated partial thromboplastin time; CRP: C-reactive protein; ESR: erythrocyte sedimentation rate; ANA: antinuclear antibody; ANCA: antineutrophil cytoplasmic antibody

Parameter	Patient value	Reference range
Hemoglobin	9.8 g/dL	12-16 g/dL
Total leukocyte count	8.4×10⁹/L	4-11×10⁹/L
Platelet count	62×10⁹/L	150-450×10⁹/L
Serum creatinine	2.3 mg/dL	0.6-1.2 mg/dL
Serum urea	59 mg/dL	7-20 mg/dL
ALT	68 U/L	7-56 U/L
AST	78 U/L	10-40 U/L
Serum albumin	2.8 g/dL	3.5-5.5 g/dL
D-dimer	>5000 ng/mL	<400 ng/mL
Fibrinogen	145 mg/dL	200-400 mg/dL
PT	14 sec	11-14 sec
INR	1.2	0.8-1.2
APTT	32 sec	25-35 sec
CRP	128 mg/L	<5 mg/L
ESR	18 mm/hr	<20 mm/hr
Serum procalcitonin	0.3 ng/mL	<0.1 ng/mL
Troponin I	Positive	Negative
Protein C	30%	70-140%
Protein S	40%	60-130%
Antithrombin III	40%	80-120%
Lupus anticoagulant	Positive	Negative
Anti-β2 glycoprotein I IgM	Negative	Negative
Anti-β2 glycoprotein I IgG	Negative	Negative
Anti-β2 glycoprotein I IgA	24.99 U/ML	<20 U/ML
Cardiolipin antibody IgM	Negative	Negative
Cardiolipin antibody IgG	Negative	Negative
ANA	Negative	Negative
ANCA	Negative	Negative
Factor V Leiden mutation	Heterozygous positive	Not detected
MTHFR mutation	Not detected	Not detected

**Figure 2:**
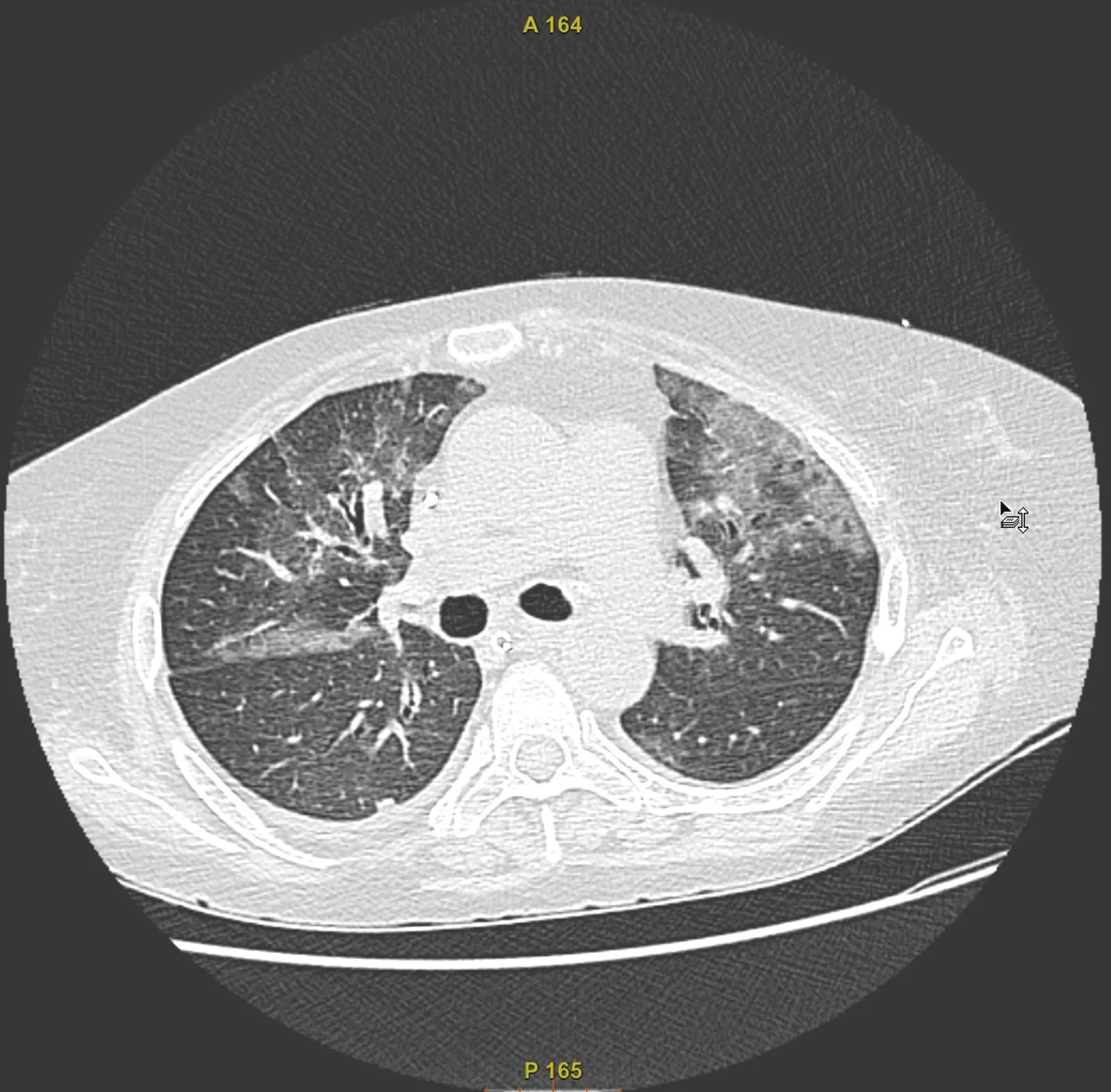
High-resolution computed tomography of the thorax showing bilateral ground-glass opacities

**Figure 3:**
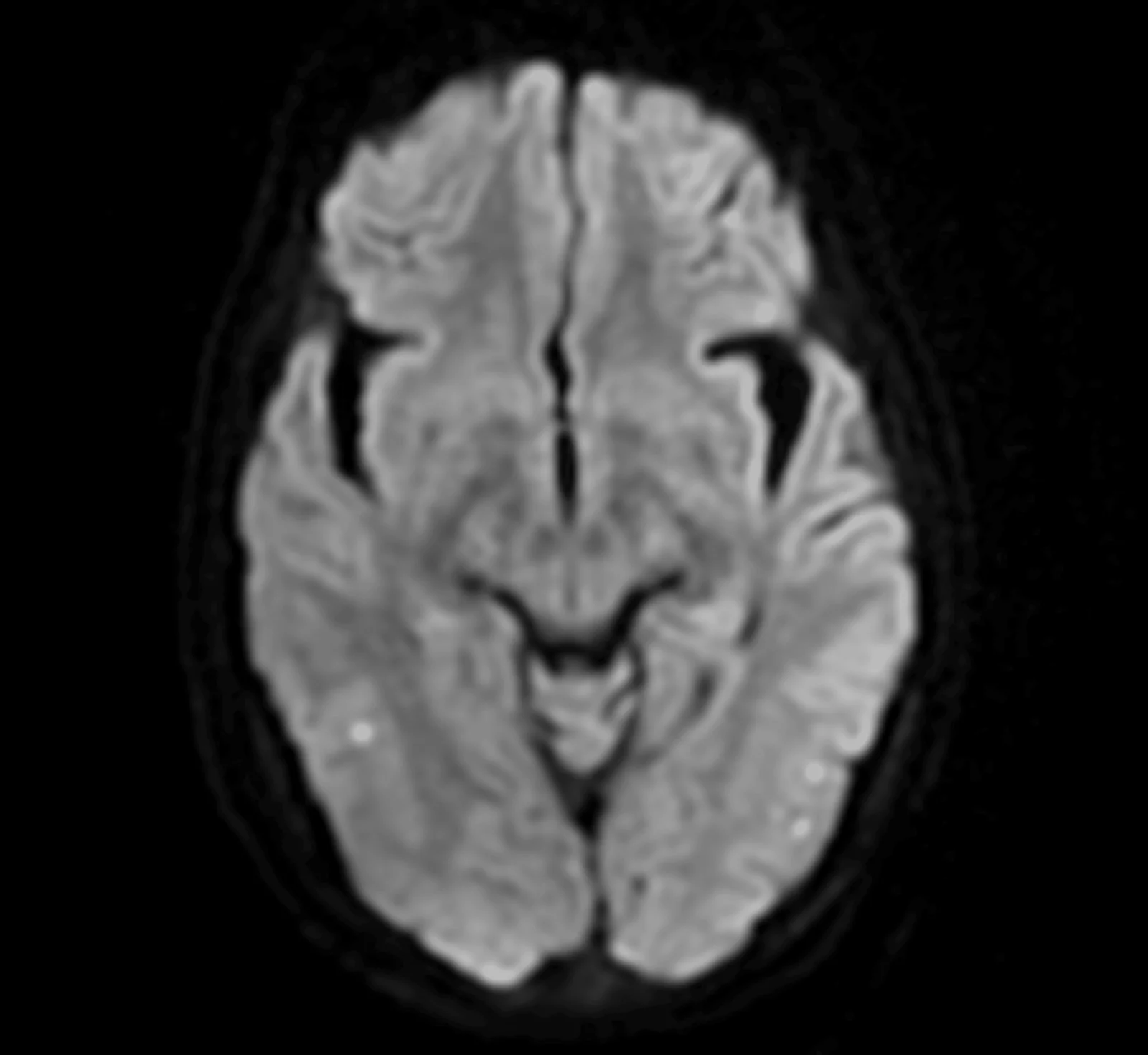
Magnetic resonance imaging of the brain (diffusion-weighted imaging) showing multiple acute lacunar infarcts consistent with small-vessel ischemic injury

**Figure 4:**
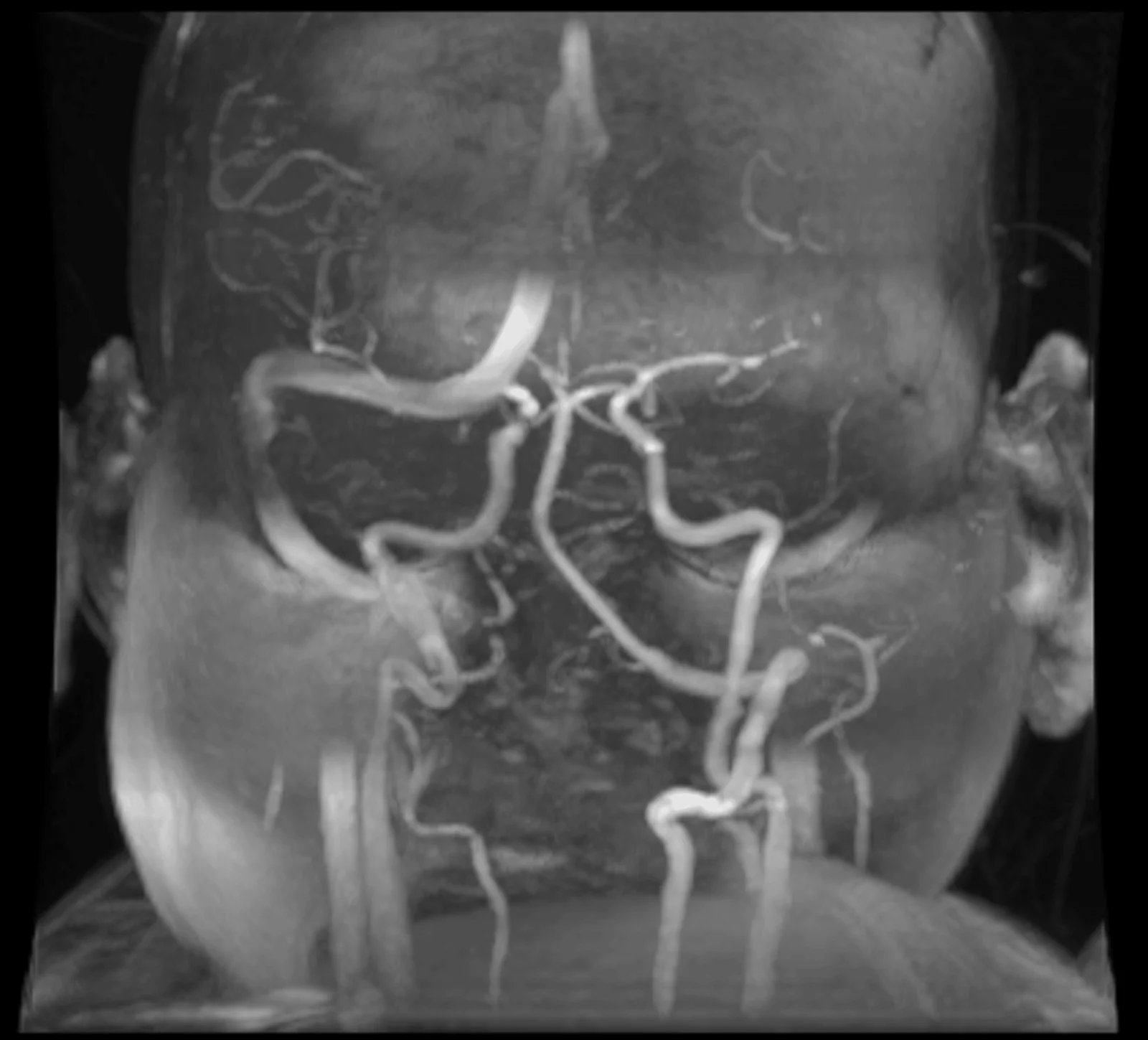
Magnetic resonance angiography showing patent major intracranial arteries without large-vessel occlusion

**Figure 5:**
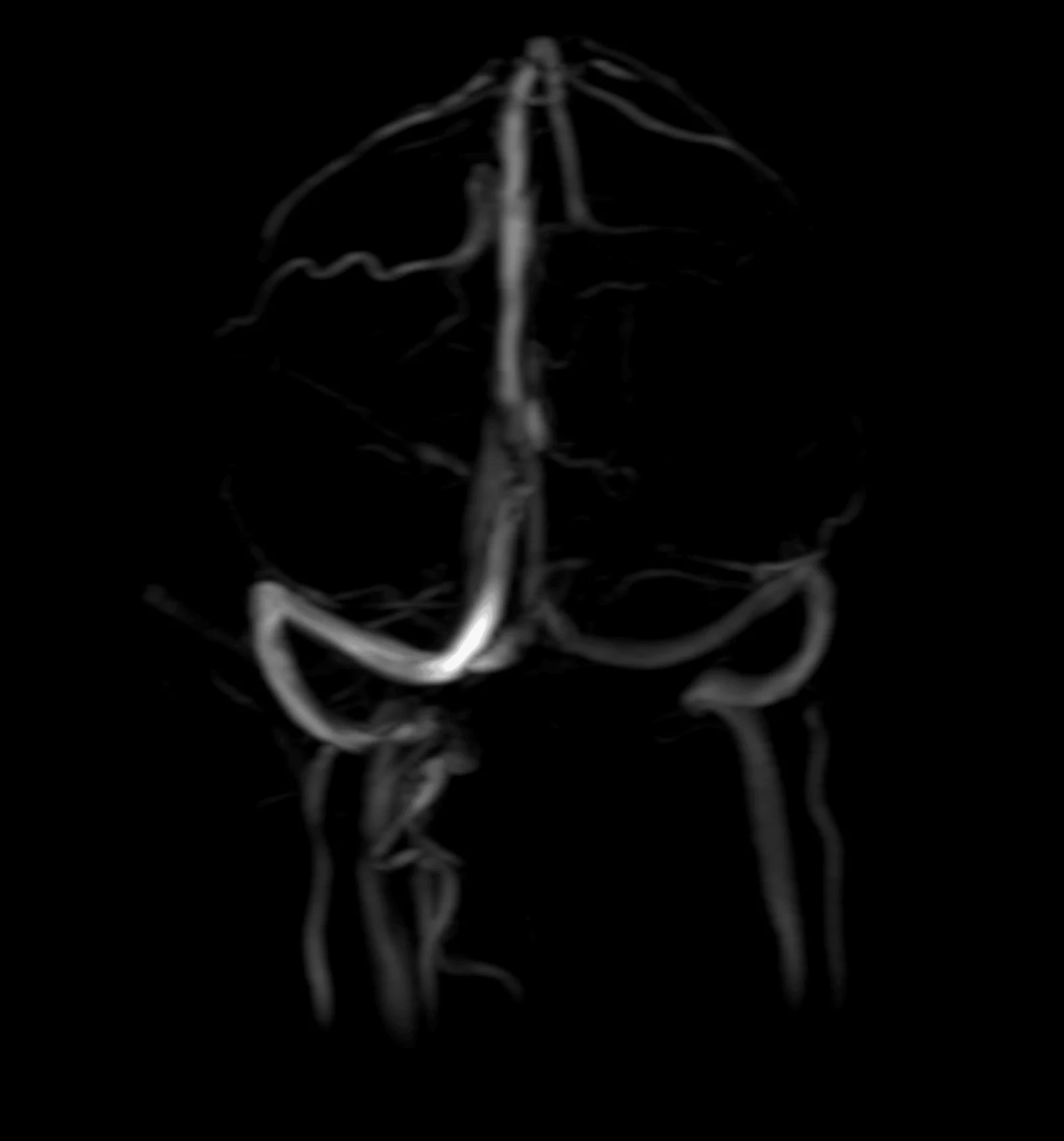
Magnetic resonance venography showing patent dural venous sinuses without thrombosis

Imaging studies revealed the following: diffuse distal arterial narrowing without large-vessel occlusion on bilateral lower limb arterial Doppler, no evidence of deep venous thrombosis on bilateral lower limb venous Doppler ultrasonography, preserved left ventricular systolic function and no regional wall motion abnormality on 2D echocardiogram, bilateral diffuse ground-glass opacities on high-resolution computed tomography (HRCT) of the chest (Figure 2), and multiple acute lacunar infarcts involving the bilateral frontoparietal and posterior temporal regions, consistent with small-vessel ischemic injury on magnetic resonance imaging (MRI) of the brain (Figure 3). Meanwhile, MR angiography (Figure 4) and venography (Figure 5) excluded major arterial or venous thrombosis.

**Figure 6:**
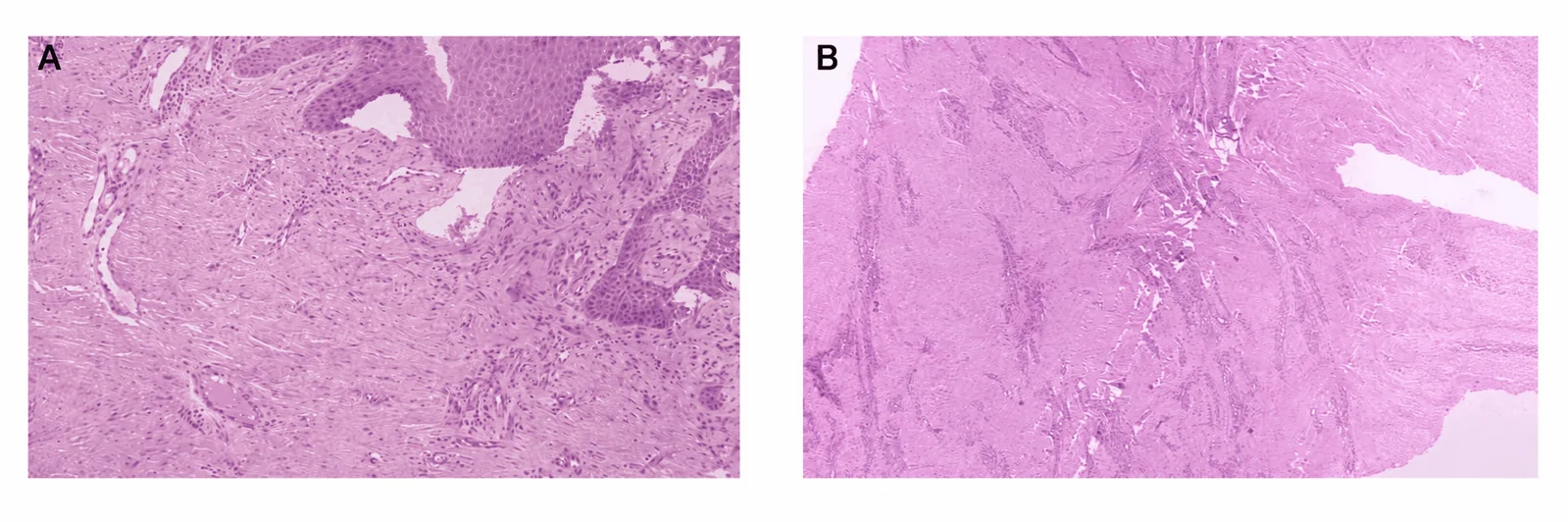
Skin biopsy (H&E stain) (A) Low-power photomicrograph (H&E, ×10) demonstrating occlusive arteriolopathy with marked intimal hyperplasia involving dermal small- to medium-sized arteries. (B) Low-power photomicrograph (H&E, ×10) demonstrating luminal thrombosis within a thickened arterial wall without fibrinoid necrosis or significant inflammatory infiltrate, supporting thrombotic microangiopathy rather than immune complex-mediated vasculitis. H&E: hematoxylin and eosin

Skin biopsy from the ischemic lesion demonstrated occlusive arteriolopathy with intimal hyperplasia and luminal thrombosis, without fibrinoid necrosis or leukocytoclastic inflammation. Direct immunofluorescence was negative for IgG, IgM, and C3, supporting a thrombotic microangiopathic process rather than immune complex-mediated vasculitis (Figure 6).

Pulmonary thromboembolism was considered in view of acute respiratory distress. However, the absence of deep venous thrombosis together with diffuse bilateral ground-glass opacities and multiorgan microvascular thrombosis favored pulmonary microvascular involvement secondary to CAPS.

Based on rapid multiorgan ischemic involvement affecting the brain, lungs, kidneys, myocardium, and peripheral circulation, confirmed lupus anticoagulant positivity, exclusion of alternative vasculitic and embolic causes, and supportive histopathology, a diagnosis of microvascular thrombotic disease consistent with CAPS was established. The heterozygous factor V Leiden mutation was considered a second-hit prothrombotic amplifier, contributing to the severity and fulminant progression of the disease.

The differential diagnoses included disseminated intravascular coagulation, thrombotic thrombocytopenic purpura, complement-mediated thrombotic microangiopathy, systemic vasculitis, infective endocarditis with embolic phenomena, and embolic arterial disease. Disseminated intravascular coagulation was considered less likely because the coagulation profile was not consistent with overt consumption coagulopathy. Systemic vasculitis was considered but was not supported by the absence of characteristic clinical features and the skin biopsy findings, which demonstrated thrombotic occlusive arteriolopathy without evidence of inflammatory vasculitis. Embolic large-vessel disease was excluded by vascular imaging demonstrating diffuse distal arterial narrowing without proximal occlusion.

The patient was treated with therapeutic unfractionated heparin infusion and high-dose corticosteroids along with aggressive supportive care, including invasive mechanical ventilation, vasopressor support, and hemodialysis. Broad-spectrum antimicrobials were administered empirically during intensive care admission. Despite adequate anticoagulation and immunomodulation, the patient demonstrated relentless progression of thrombotic organ injury with worsening respiratory failure, progressive renal dysfunction, myocardial injury, and advancing peripheral ischemia.

During the terminal phase of hospitalization, after prolonged mechanical ventilation and irreversible multiorgan damage, the patient developed a hospital-acquired infection. This event acted as a terminal physiological stressor, precipitating refractory shock. She developed sudden bradycardia progressing to pulseless electrical activity. Cardiopulmonary resuscitation was initiated promptly but was unsuccessful.

CAPS remains a clinical diagnosis, and delays in initiating therapy while awaiting confirmatory investigations may be fatal. Early anticoagulation and immunomodulatory treatment are critical to improving survival.

## Discussion

CAPS represents the most severe phenotype of APS and is characterized by rapidly progressive small-vessel thrombosis affecting multiple organs within a short time span, typically less than one week [2,4]. Unlike classical APS, in which large-vessel thrombosis predominates, CAPS is primarily a microvascular thrombotic process, leading to widespread organ dysfunction, including renal failure, pulmonary involvement, neurological deficits, and cutaneous ischemia [2].

The pathogenesis of CAPS is thought to involve a "two-hit" mechanism, wherein the presence of antiphospholipid antibodies primes the endothelium and coagulation cascade, while an additional trigger, such as infection, surgery, trauma, or inflammatory stress, precipitates an uncontrolled thrombotic cascade [2]. Lupus anticoagulant remains the strongest laboratory predictor of thrombotic risk and is frequently positive in CAPS [1]. In our patient, lupus anticoagulant positivity in the setting of thrombocytopenia, elevated D-dimer, reduced fibrinogen, and multiorgan dysfunction strongly supported a thrombotic microangiopathic process. The presence of a heterozygous factor V Leiden mutation may have acted as a prothrombotic amplifier, consistent with the second-hit hypothesis [3].

Neurological manifestations in CAPS are common and may present as multiple small infarcts rather than large territorial strokes [2]. The MRI findings in this case demonstrated multiple acute lacunar infarcts without major arterial occlusion, consistent with diffuse microvascular thrombosis. Similarly, pulmonary ground-glass opacities likely reflected pulmonary microvascular involvement rather than primary infection. Recognition of this pattern is crucial, as imaging may misleadingly suggest alternative etiologies.

The current recommended management of CAPS includes prompt initiation of therapeutic anticoagulation, high-dose corticosteroids, and, in selected cases, plasma exchange and/or intravenous immunoglobulin [2,5]. Combination therapy has been associated with improved survival compared with anticoagulation alone [5]. Despite advances in management, mortality remains high, ranging from 30% to 50% in reported series, particularly in cases with delayed diagnosis or multiorgan failure at presentation [2,6].

This case underscores the importance of early clinical suspicion. In rapidly deteriorating patients with laboratory evidence of consumptive coagulopathy and positive lupus anticoagulant, treatment should not be delayed pending complete antibody panels or repeat confirmatory testing. CAPS remains fundamentally a clinical diagnosis supported by laboratory findings rather than one dependent solely on strict classification criteria [2,7].

## Conclusions

CAPS should be considered in patients presenting with rapidly progressive multiorgan ischemia and laboratory evidence of a prothrombotic state, even in the absence of prior thrombotic events, obstetric manifestations, or established autoimmune disease. This case highlights the diagnostic value of integrating clinical presentation, imaging, serological testing, and histopathology to differentiate CAPS from other thrombotic and vasculitic disorders. Early recognition and prompt initiation of anticoagulation and immunomodulatory therapy remain essential to optimize outcomes in this rapidly progressive and often fatal condition.
